# Phenyllactic acid promotes cell migration and invasion in cervical cancer via IKK/NF-κB-mediated MMP-9 activation

**DOI:** 10.1186/s12935-019-0965-0

**Published:** 2019-09-23

**Authors:** Chao Li, Yanfei Li, Lanxia Sui, Jian Wang, Fang Li

**Affiliations:** 1 Clinical and Translational Research Center, Shanghai First Maternity and Infant Hospital, Tongji University School of Medicine, No. 2699, West GaoKe Road, Shanghai, 201204 China; 2 School of Medical Technology, Shanghai University of Medicine & Health Sciences, Shanghai, 201318 China; 3 Department of Pediatrics, PLA No. 904 Hospital, Wuxi, 214000 China; 4 School of Medicine, Shanghai Jiao Tong University, Shanghai, 200025 China; 5 Department of Gynecology, Shanghai First Maternity and Infant Hospital, Tongji University School of Medicine, No. 2699, West GaoKe Road, Shanghai, 201204 China

**Keywords:** Phenyllactic acid, Human papillomavirus, Cervical cancer, Migration and invasion, NF-κB

## Abstract

**Background:**

Persistent infection with high-risk human papillomavirus (hrHPV) is associated with cervical cancer development. This process involves the virus-encoded E6 and E7 oncoproteins, which are maintained and expressed during all malignant transformation stages. However, HPV alone is insufficient to drive tumor progression-related behaviors such as cervical cancer cell motility. In this study, we investigated the effect of phenyllactic acid (PLA), a phenolic acid phytochemical and biomarker for discriminating various cancers, on the metastatic potential of cervical cancer cells.

**Methods:**

The effects of PLA on HPV16/18 E6/E7 expression, migratory and invasive behavior, and matrix metalloproteinases (MMPs) expression of cervical cancers cells were measured. Specific inhibitors were used to further investigate biological function and underlying mechanism of PLA modulated cell motility.

**Results:**

PLA significantly promoted the migration and invasion of SiHa, HeLa, and C-33A cervical cancer cells as well as upregulated matrix metalloproteinase-9 (MMP-9) expression. Moreover, PLA treatment attenuated E6/E7 expression in SiHa and HeLa cells. Further molecular analysis showed that PLA activated the nuclear factor-kappa B (NF-κB) signaling pathway and increased the nuclear translocation of both IκBα and p65. Treating cervical cancer cells with an NF-κB inhibitor potently reversed PLA-induced migratory and invasive behavior, MMP-9 upregulation, and/or E6/E7 downregulation. The PLA-induced NF-κB activation and MMP-9 upregulation were mediated by IκB kinase-β (IKK-β) phosphorylation via PKC signals. The results suggested that SiHa, HeLa, and C-33A cells might undergo a similar process to enhance their motility in response to PLA, regardless of the HPV status.

**Conclusions:**

Collectively, our study reveals a new biological function of PLA and elucidate the possible molecular role of PLA as a risk factor for triggering cervical cancer cell motility.

## Background

Persistent infection with specific human papillomaviruses (HPVs), such as high-risk (hr) HPV16 and HPV18, underlie the development of approximately 70% of cervical cancers [1]. The E6 and E7 proteins are the two major transforming proteins encoded by oncogenic HPVs, and the consistent expression of these two oncoproteins can immortalize human cervical epithelial cells, thus inducing the development of precancerous lesions, which could progress to cervical cancer if left untreated [2]. HPV E6 and E7 function by targeting host pathways that modulate many downstream effectors, thus resulting in alterations in relevant physiological processes that are considered hallmarks of cancer [3]. Notably, the HPV E6 and E7 proteins bind and target the tumor suppressor proteins p53 and pRB, respectively, for proteasomal degradation [4]. In addition, E6 and E7 interact with and regulate the function of many other multifunctional proteins, including transcription factors and epigenetic regulators, which in turn causes alterations in cellular gene expression [5]. However, the underlying molecular mechanism of HPV-induced cervical cancer remains incompletely understood.

*Lactobacillus* is the predominant bacteria in healthy vagina and plays an vital role in protection of female reproduction system. It exerts its protective functions mainly via several mechanisms, including preventing pathogenic bacteria by acting as a barrier on the epithelial cells, activating the immune system, and secreting various metabolites and organic acid [6]. Phenyllactic acid (PLA), which is a phenolic acid phytochemical, is most frequently produced by *Lactobacillus*. PLA has gained increased attention due to its broad spectrum of antimicrobial activity against gram-positive and gram-negative bacteria as well as fungi [7]. Animal experiments suggest that PLA as a dietary supplement for animals could reduce the number of coliform bacteria and improve immune characteristics [8]. Moreover, the antimicrobial activity of PLA is better and its odor is less potent than those of other organic acids, such as acetic acid and lactic acid [9]. In previous studies, PLA was reported to be a major metabolic biomarker for lipid oxidative damage to the cerebral cortex, phenylketonuria, and alcohol-induced liver disease [10]. More recently, PLA was recognized as a potential biomarker to identify patients with ovarian cancer [11], oral squamous cell carcinoma, and cervical cancer [12]. Although PLA was well documented to play a positive role during the progression of different cancers, its potential molecular mechanism has not been validated.

To elucidate the above two points, this study used cervical cancer as an experimental model and aimed to investigate how PLA contributes to the development of cervical cancer by using HPV-positive SiHa (HPV16) and HeLa (HPV18) cell lines and the HPV-negative C-33A cell line as the control. We first examined the influence of extracellular PLA on the expression of HPV16/18 E6 and E7 in SiHa and HeLa cells as well as on the migratory and invasive behavior of SiHa, HeLa, and C-33A cells. Then, downstream signaling pathways in the PLA-induced migration and invasion of cervical cancer cells were evaluated. The results showed that PLA could modulate HPV16/18 E6 and E7 expression and increase cell motility by upregulating MMP-9 via activation of the IKK/NF-κB signaling pathway.

## Materials and methods

### Materials and chemicals

The restriction enzymes and T_4_ DNA ligase were purchased from New England Biolabs (Beverly, MA). DL-3-phenyllactic acid (PLA, ≥ 98.0%) was obtained from Sigma-Aldrich (St. Louis, MO, USA). Oligonucleotide biosynthesis was carried out by Generay Biotech Co., Ltd. (Shanghai, China). BAY11-7082 (BAY, an inhibitor of NF-κB), PD98059 (PD, a specific antagonist of ERK1/2 kinase), LY294002 (LY, a selective antagonist of PI3K/Akt), AG1478 (AG, a potent antagonist of EGFR), and H89 (H89, a selective antagonist of PKA) were purchased from MedChem Express (MedChem Express, China). IMD0354 (IMD, an inhibitor of IKK-β) and GF109203X (GF, a specific antagonist of PKC) were purchased from Selleck Chemicals (Selleck Chemicals, China). An NE-PER™ Nuclear and Cytoplasmic Extraction Reagents kit was purchased from Pierce. All the other chemicals and reagents were of at least analytical grade and were available commercially.

### Cell culture

The HPV16- and HPV18-positive human cervical cancer cell lines SiHa and HeLa and the HPV-negative human cervical cancer cell line C-33A were purchased from the American Type Culture Collection and were authenticated by an short tandem repeat (STR) test. SiHa/C-33A and HeLa cells were respectively cultured in MEM and DMEM (HyClone, Logan, UT) supplemented with 10% fetal bovine serum (FBS, Gibco, NY, USA) and 1% penicillin–streptomycin (P/S, Gibco, NY, USA). Cells were grown in a humidified 5% CO_2_ incubator at 37 °C. SiHa, HeLa, or C-33A cells (2 × 10^5^ cells per well) were incubated in 6-well plates to 70–80% confluence and were then treated with or without PLA at a concentration ranging from 2.5 to 20 mM. The culture medium was removed after treatment, and the monolayers were washed with phosphate-buffered saline (PBS) and then immediately used for total RNA and protein extraction.

### RNA extraction and qRT-PCR analysis of mRNA

Total RNA was extracted from untreated cells and from cells treated with PLA with TRIzol reagent (Invitrogen) and was then incubated with DNase I. A 0.5 µg sample of total RNA was used for cDNA synthesis with a Two-Step PrimeScript miRNA cDNA Synthesis Kit (Takara, Dalian, China) and an ABI 7500 Real-Time PCR system (Applied Biosystems, Foster City, CA, USA). qRT-PCR was carried out with a SuperReal PreMix Plus (SYBR Green) Kit (TIANGEN Biotech Co., Ltd, Beijing, China) using a Light Cycler system. The relative RNA expression level after normalization to GAPDH was calculated according to the change in expression using the equation 2^−ΔΔCt^. The primers used for the qRT-PCR analysis are listed in Additional file 1: Table S1.

### Western blot analysis

Total lysates from PLA-treated SiHa, HeLa, or C-33A cells were prepared in RIPA lysis buffer supplemented with a protease inhibitor cocktail (Roche, Basel, Switzerland) and phenylmethylsulfonyl fluoride (Sigma-Aldrich, St. Louis, MO, USA). Equal amounts of protein were separated by sodium dodecyl sulfate (SDS)-polyacrylamide gel electrophoresis and blotted onto polyvinylidene difluoride membranes (Millipore, Billerica, MA, USA), which were then blocked in 5 or 7% skim milk for 1 or 2 h at room temperature. Blots were incubated with the indicated primary antibodies against HPV16/18 E6 (ab70), HPV16 E7 (ab30731), and HPV18 E7 (ab100953) (Abcam, London, UK) and against MMP-1 (BS1229), MMP-2 (BS1236), MMP-3 (BS6243), MMP-9 (BS1241), MMP-10 (BS7167), MMP-13 (BS6668), IκBα (BS1190), p-IκBα (phospho-S32/S36, BS4105), p65 (BS1253), p-p65 (phospho-S536, BS4138), IKK-β (BS1756), and p-IKK-β (phospho-S180/181, BS4237) (Bioworld Technology, Nanjing, China) at 4 °C overnight, followed by incubation with a goat anti-rabbit (ab6721) or rabbit anti-mouse (ab6728) IgG secondary antibody (Abcam, London, UK) for 1 h at room temperature. An anti-GAPDH antibody (sc-32233) (Santa Cruz Biotechnology, CA, USA) was used as a loading control. Immunocomplexes were detected with an enhanced chemiluminescence (ECL) Kit (MilliporeSigma, Burlington, MA, USA) and visualized with a FluorChem E imaging instrument (ProteinSimple, San Jose, CA, USA).

### Subcellular localization of p65

Nuclear and cytoplasmic extracts from PLA-treated SiHa, HeLa, or C-33A cells were prepared using NE-PER™ nuclear and cytoplasmic extraction reagents (Pierce) according to the manufacturer’s instructions. Twenty micrograms of total protein from each preparation was separated by SDS-PAGE, followed by immunoblotting with an anti-p-p65 antibody to detect the phosphorylation of p65 protein. Anti-histone H2A (BS1499) (Bioworld Technology, Nanjing, China) and GAPDH antibodies were used as loading controls for the expression of nuclear and cytoplasmic proteins, respectively.

### NF-κB luciferase reporter assay

The luciferase reporter plasmid pNF-κB-Luc contains four repeats of κB binding motifs at the *Nhe*I/*Bgl*II restriction site followed by the luciferase reporter gene (Luc). For the luciferase reporter activity assay, HeLa and C-33A cells were plated in 24-well plates at a density of 4.5 × 10^4^ cells per well and grown overnight. Cells were transfected with a mixture of 250 ng of the NF-κB promoter construct pNF-κB-Luc and 5 ng of the pRL-TK control vector (Promega, Madison, WI) using Lipofectamine 2000. After 24 h in culture, a dual-luciferase reporter assay system (Promega, WI, USA) was used according to the manufacturer’s instructions.

### Transwell migration and invasion assays

Transwell migration and invasion assays were carried out using a chamber containing a polycarbonate filter with a pore size of 8.0 μm (24-well insert; Corning, NY, USA). Polycarbonate filters precoated with Matrigel Matrix (BD Biosciences, San Jose, CA, USA) were used for the invasion assay, and uncoated filters were used for the migration assay. Cells were serum-starved for 8 h. The lower chambers were filled with 800 mL of MEM (SiHa/C-33A) or DMEM (HeLa) supplemented with (concentrations of 2.5–20 mM) or without PLA. Approximately 3–5 × 10^4^ cells were resuspended in 300 μL of serum-free medium and added to the upper chamber. After incubation for 16 h at 37 °C and 5% CO_2_, the lower chambers were incubated with calcein-AM (Thermo Fisher Scientific) to stain the cells that invaded through the uncoated membrane or the Matrigel Matrix. The stained cells were counted in 4 randomly selected visual fields using a CCD camera mounted to an inverted microscope running MetaMorph image analysis software (Molecular Devices, Sunnyvale, CA).

### Immunofluorescence staining

Cell suspensions were seeded on the central concavity of a special 35 mm glass bottom plate (Nest, China) at ~ 50% confluent density. The cells were cultured overnight with or without PLA. After three washes with ice-cold PBS, cells were fixed with 4% paraformaldehyde for 15 min and then permeabilized with 0.1% Triton X-100 for 10 min. Subsequently, cells were blocked with 5% bovine serum albumin for 1 h at room temperature and incubated with an appropriate concentration of primary antibodies against Oct4, ABCG2, ALDH1, SOX2, or CD49f (Abcam, London, UK, 1:100 dilution) overnight at 4 °C. Then, the cells were incubated with Alexa Fluor^®^ 488 conjugated goat anti-rabbit IgG (Abcam, London, UK, 1:200 dilution) or goat anti-mouse IgG (Abcam, London, UK, 1:200 dilution) secondary antibodies for 1 h. The stained cells were observed under a fluorescence confocal microscope.

### Statistical analysis

Graphical data are presented as the means ± standard deviations (SDs) of three independent experiments. Statistical analysis was performed using GraphPad Prism 7.0 (GraphPad Software, San Diego, CA, USA) with one-way analysis of variance (ANOVA) followed by Bonferroni’s post hoc test. A *P*-value < 0.05 compared with the control was considered statistically significant.

## Results

### PLA inhibited E6/E7 expression and promoted the migration and invasion of cervical cancer cells

**Fig. 1:**
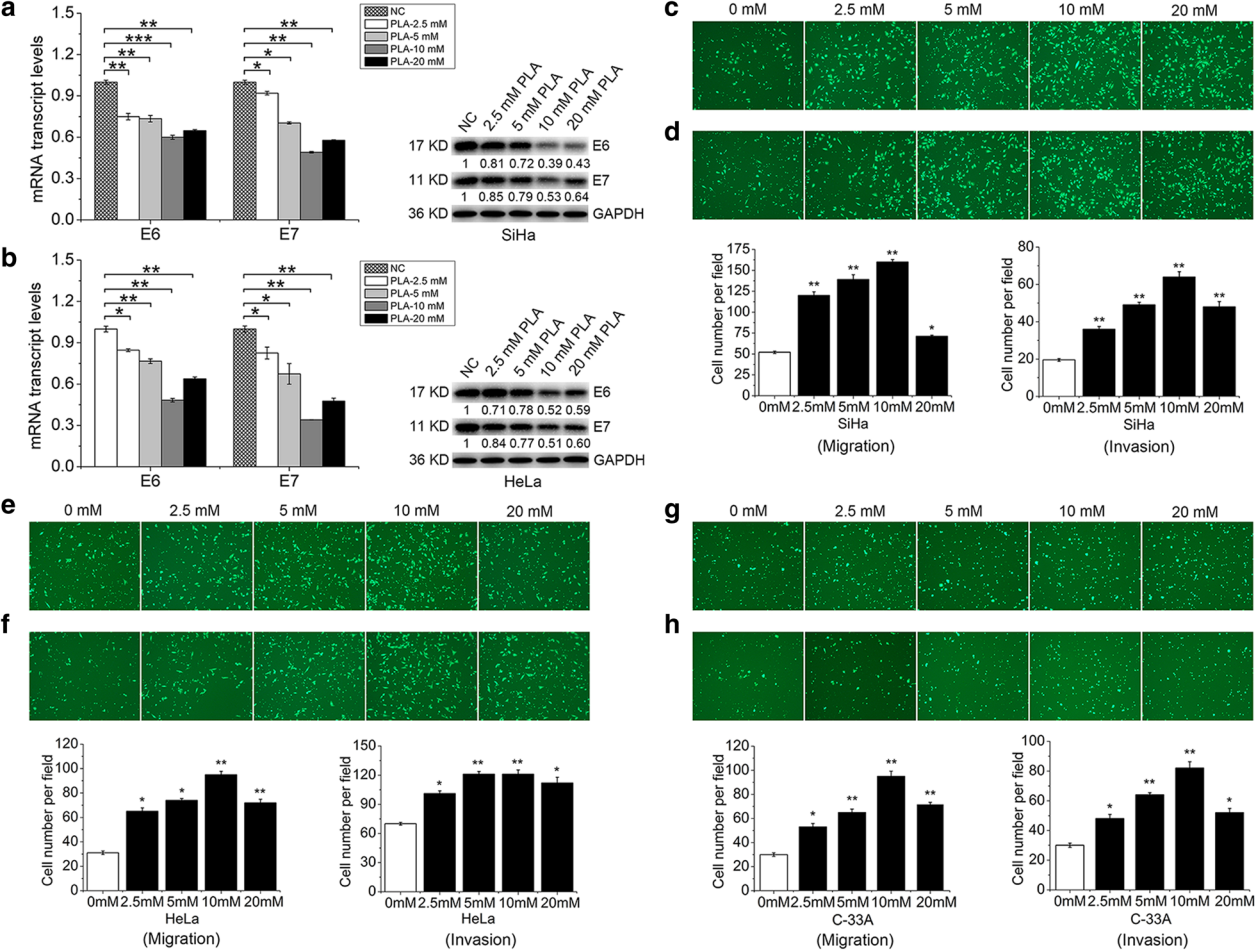
Effects of PLA on HPV E6 and E7 expression and on the migration and invasion of cervical cancer cells. **a**,  **b** The transcriptional and translational levels of E6 and E7 in SiHa (**a**) and HeLa (**b**) cells treated with different concentrations of PLA (2.5, 5, 10, and 20 mM) for 24 h. GAPDH expression was evaluated as a loading control. One representative of three different experiments, for each of the analyses performed, is shown. **c**, **d** Effects of different concentrations of PLA (2.5, 5, 10, and 20 mM) on the migration (**c**) and invasion (**d**) of SiHa cells as determined by Transwell assay. **e**, **f** Effects of different concentrations of PLA (2.5, 5, 10, and 20 mM) on the migration (**e**) and invasion (**f**) of HeLa cells as determined by Transwell assay. **g**, **h** Effects of different concentrations of PLA (2.5, 5, 10, and 20 mM) on the migration (**g**) and invasion (**h**) of C-33A cells as determined by Transwell assay. Data are presented as the means ± SDs of three independent experiments. ^*^P < 0.05, ^**^P < 0.01, and ^***^P < 0.001 compared with the control

To assess whether PLA has functional roles in the regulation of E6 and E7 expression in HPV16/18-integrated SiHa and HeLa cells or HPV-negative C-33A cells, racemic DL-PLA was used at a concentration ranging from 2.5 to 20 mM, which is defined as a nontoxic concentration [13]. The qRT-PCR analysis showed that PLA significantly suppressed E6 and E7 mRNA expression in both SiHa and HeLa cell lines (Fig. 1a, b). A concentration of 10 mM PLA induced a substantially greater decrease in E6 and E7 mRNA than other PLA concentrations. The subsequent Western blot analysis confirmed the inhibitory effects of PLA on E6 and E7 protein levels observed in the qRT-PCR analysis.

Then, SiHa, HeLa, and C-33A cell migration and invasion were assessed by Transwell assays to test the effect of PLA on cervical cell motility. Compared to those of control cells, exogenously adding PLA led to a significant promotion of migration (Fig. 1c, e, g) and invasion (Fig. 1d, f, h) in all three cell lines. No visible difference was found between the HPV-positive SiHa/HeLa and HPV-negative C-33A cells. However, 10 mM PLA more potently increased the number of invaded cells by at least 2.1-fold than other PLA concentrations. Furthermore, we performed proliferation experiments to investigate the effects of PLA on SiHa, HeLa, and C-33A cells under the same culture conditions. The experiments showed that treatment with 2.5 to 20 mM PLA for 72 h did not induce notable growth inhibitory effects affecting the proliferation of the three cell lines (Additional file 1: Fig. S1). Thus, our data suggested that millimolar concentrations of PLA can inhibit the expression of the HPV16/18 E6 and E7 oncoproteins and promote the in vitro migration and invasion of SiHa, HeLa, and C-33A cells. Accordingly, a concentration of 10 mM PLA was chosen for the subsequent experiments.

### PLA upregulated the expression of migration- and invasion-related proteins in cervical cancer cells

**Fig. 2:**
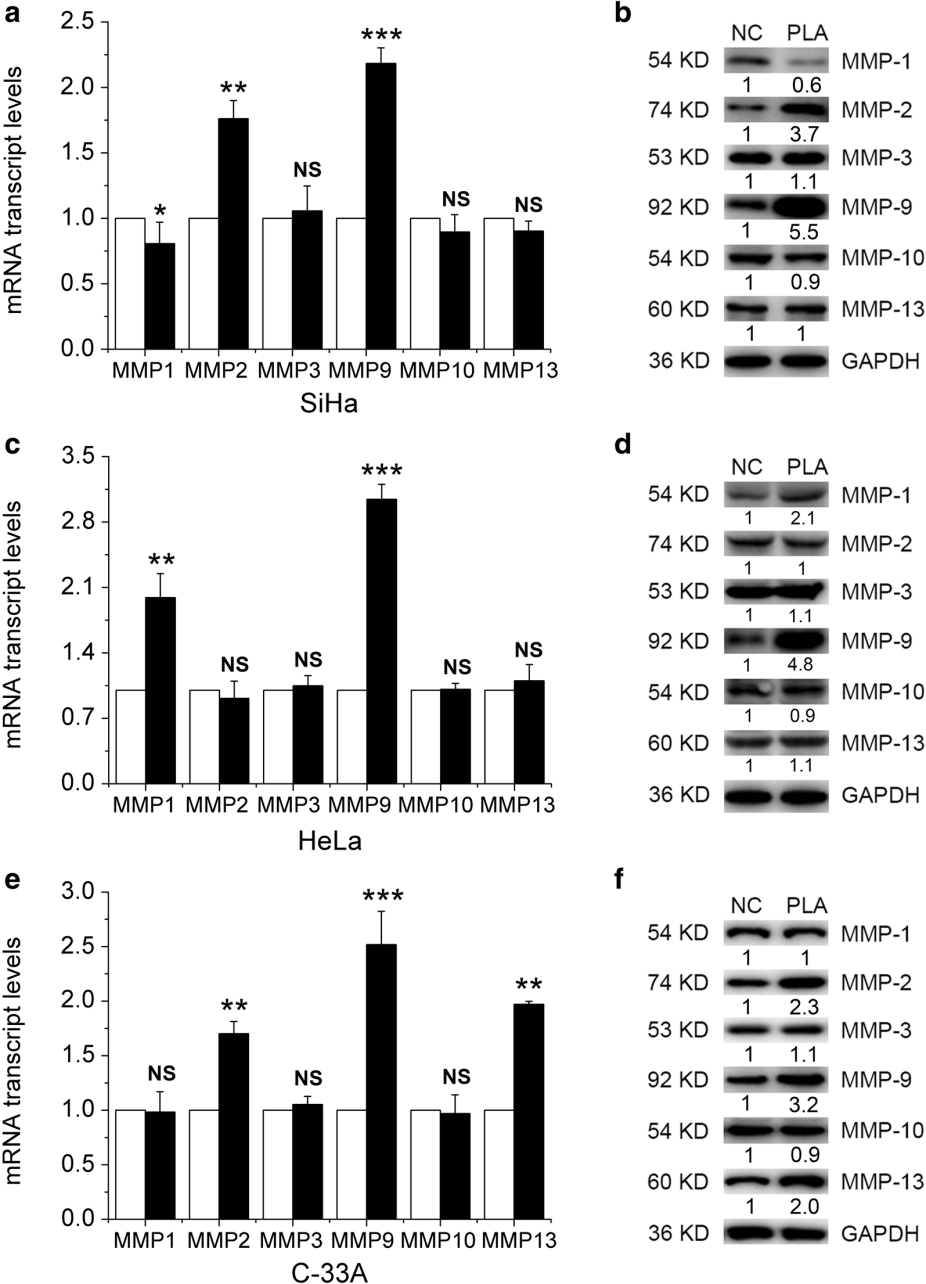
Effects of PLA on the expression of migration- and invasion-related proteins in cervical cancer cells. MMP expression was detected by real-time PCR analysis in SiHa (**a**), HeLa (**c**), and C-33A (**e**) cells treated with and without PLA (10 mM) for 24 h. Data are presented as the means ± SDs of three independent experiments. ^*^P < 0.05, ^**^P < 0.01, and ^***^P < 0.001 compared with the control. After treatment with PLA for 24 h, the expression of MMP-1, MMP-2, MMP-3, MMP-9, MMP-10 and MMP-13 in SiHa (**b**), HeLa (**d**), and C-33A (**f**) cells was detected by Western blot analysis. GAPDH expression was evaluated as a loading control. One representative of three different experiments, for each of the analyses performed, is shown

Matrix metalloproteinases (MMPs) play important roles in the degradation of the extracellular matrix, which is strongly implicated in the invasion and metastasis of malignant tumor cells [14]. Hence, the effects of PLA on the expression of MMP-1, MMP-2, MMP-3, MMP-9, MMP-10, and MMP-13 were analyzed in SiHa, HeLa, and C-33A cells. As shown in Fig. 2, treatment with 10 mM PLA significantly increased the levels of MMP-2 and MMP-9 in SiHa (Fig. 2a) cells, the levels of MMP-1 and MMP-9 in HeLa (Fig. 2c) cells, and the levels of MMP-2, MMP-9 and MMP13 in C-33A (Fig. 2e) cells. No pronounced increase in the effect of PLA on the expression of other MMPs was found in either of the three cell lines. Furthermore, the protein levels of MMPs secreted by SiHa, HeLa, and C-33A cells were examined by Western blot analysis. The results showed that PLA could significantly increase the expression of MMP-2 and MMP-9 in SiHa (Fig. 2b) cells, the expression of MMP-1, MMP-9 and MMP-13 in HeLa (Fig. 2d) cells, and the expression of MMP-2, MMP-9 and MMP-13 in C-33A (Fig. 2f) cells. Taken together, these results indicate that PLA can upregulate the expression of migration- and invasion-related MMPs in cervical cancer cells, particularly the expression of MMP-9. This upregulation in MMP expression might have enhanced the motility of these cells because the invasion of tumor cells is mainly dependent on the degradation of extracellular matrix by MMP-2 and MMP-9 [15].

### NF-κB mediated the PLA-induced migration and invasion of cervical cancer cells

**Fig. 3:**
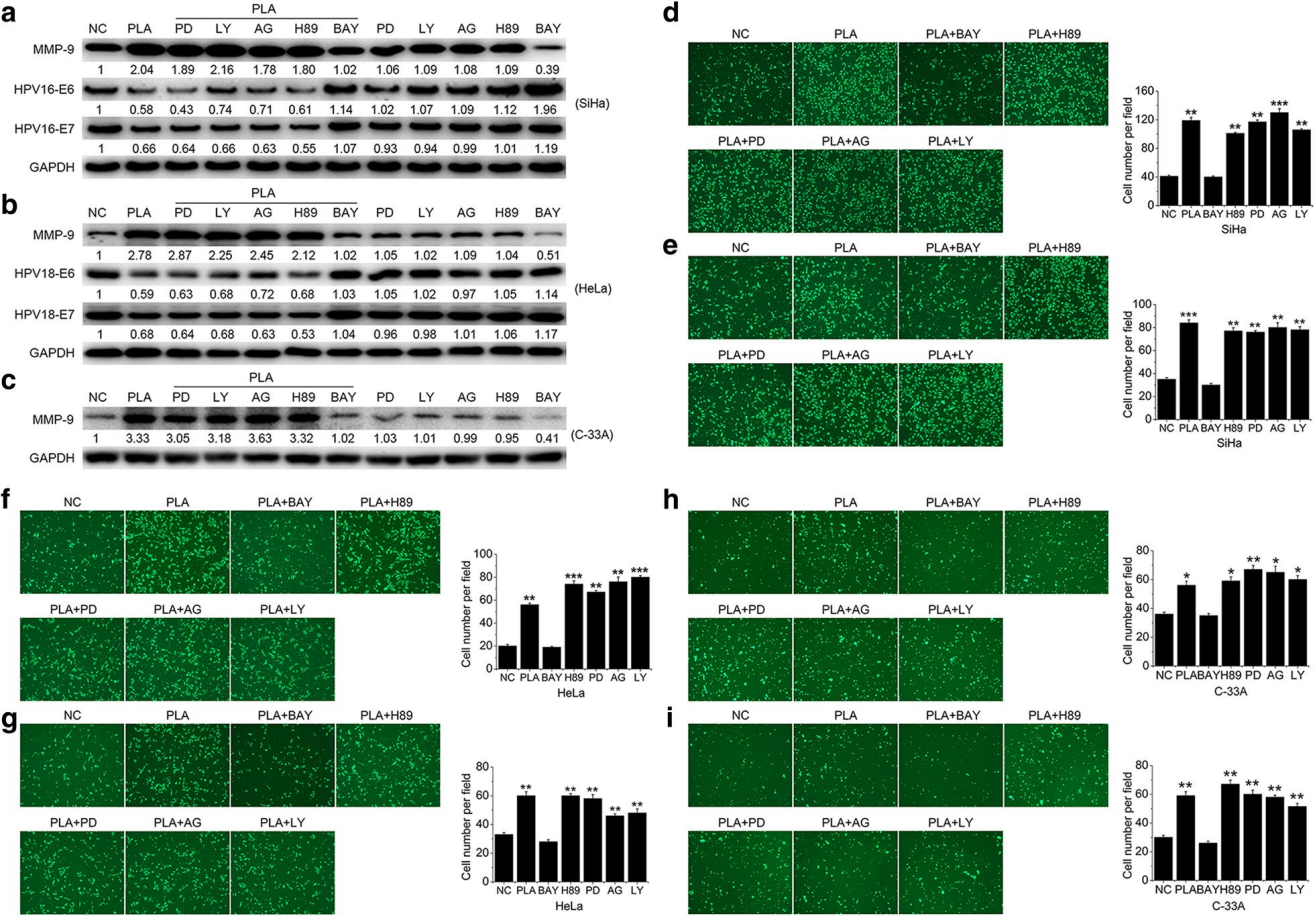
Effects of NF-κB on the PLA-induced migration and invasion of cervical cancer cells. **a**‒**c** SiHa (**a**), HeLa (**b**), and C-33A (**c**) cells were pretreated with 10 μM PD98059 (PD, a specific antagonist of ERK1/2 kinase), LY294002 (LY, a selective antagonist of PI3 K/Akt), AG1478 (AG, a potent antagonist of EGFR), H89 (H89, a selective antagonist of PKA), and BAY11-7082 (BAY, an inhibitor of NF-κB) for 90 min and then stimulated with 10 mM PLA for another 24 h. The expression of E6 and E7 (for SiHa and HeLa) and MMP-9 (for SiHa, HeLa, and C-33A) was measured by Western blot analysis. GAPDH expression was evaluated as a loading control. One representative of three different experiments, for each of the analyses performed, is shown. **d**‒**i** SiHa, HeLa, and C-33A cells were pretreated with the above inhibitors for 90 min and then stimulated with 10 mM PLA for another 24 h. Then, cell migration (**d**, **f**, **h**) and invasion (**e**, **g**, **i**) were monitored by Transwell assays. The number of migrated and invaded cells was obtained by comparison with the control cells. Data are presented as the means ± SDs of three independent experiments. ^*^P < 0.05, ^**^P < 0.01, and ^***^P < 0.001 compared with the control

Recent studies have suggested that the NF-κB, ERK1/2, PI3 K/Akt, EGFR, and PKA signaling pathways are important in the migration and invasion of cervical cancer cells [16]. To verify which signaling pathway is responsible for the PLA-induced migration and invasion of SiHa, HeLa, and C-33A cells, the regulatory mechanisms of MMP-9 and/or E6 and E7 were investigated by treatment with a specific inhibitor of each signaling pathway. As shown in Fig. 3a–c, BAY 11-7082, an inhibitor of NF-κB, selectively abolished the PLA-induced downregulation of E6 and E7 in both SiHa and HeLa cells, as well as the upregulation of MMP-9 in all three cells. Meanwhile, the inhibitors of ERK1/2 (PD 98059), PI3 K/Akt (LY 294002), EGFR (AG 1478), and PKA (H 89) had no notable effect on the expression of either E6/E7 or MMP-9. To confirm the results, these inhibitors were further used to analyze their potential effects on cell migration and invasion. It was observed that only BAY 11-7082 attenuated PLA-induced migration (Fig. 3d, f, h) and invasion (Fig. 3e, g, i) in all three cells (Additional file 1: Fig. S2). Collectively, the results indicated that NF-κB probably participated in the PLA-induced migration and invasion of cervical cancer cells.

### PLA increased NF-κB activation in cervical cancer cells

**Fig. 4:**
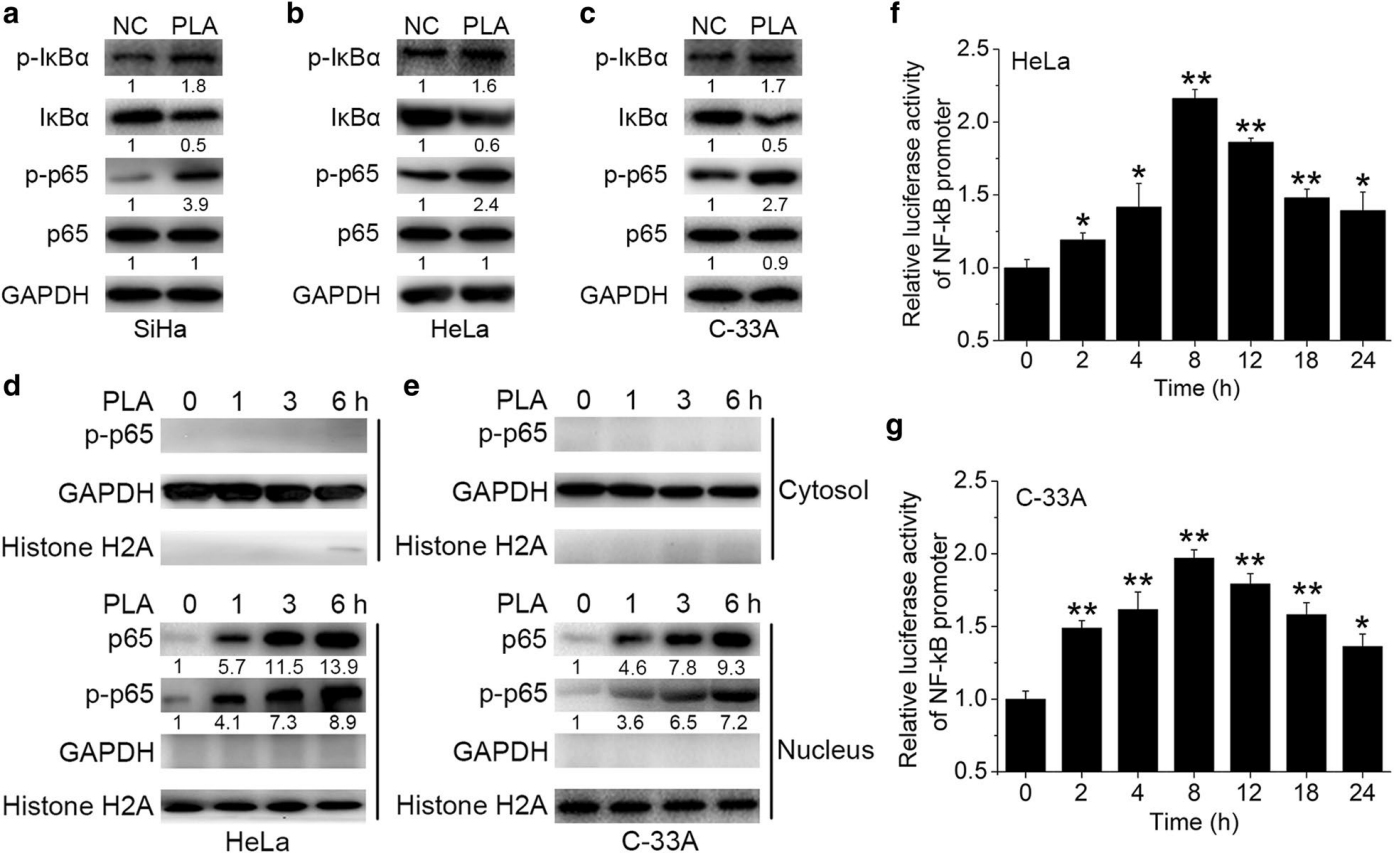
Effects of PLA on NF-κB activation in cervical cancer cells. **a**‒**c** SiHa (**a**), HeLa (**b**), and C-33A (**c**) cells were treated with 10 mM PLA for 30 min, and the activation of IκBα and p65 was then examined by Western blot analysis. GAPDH expression was evaluated as a loading control. One representative of three different experiments, for each of the analyses performed, is shown. **d**, **e**) After treatment with 10 mM PLA for the indicated times, the nuclear and cytoplasmic cellular fractions of HeLa (**d**) and C-33A (**e**) cells were isolated by differential lysis. The levels of p-p65 in the nuclear and cytoplasmic cellular fractions were detected by Western blot analysis. GAPDH and Histone H2A were used as loading controls. One representative of three different experiments, for each of the analyses performed, is shown. **f**, **g** HeLa (**f**) and C-33A (**g**) cells were cotransfected with pNF-κB-Luc and pRL-TK. After transfection for 6 h, cells were treated with 10 mM PLA for the indicated times. NF-κB reporter activity was calculated as the ratio of the activities of pNF-κB-Luc and pRL-TK. Data are presented as the means ± SDs of three independent experiments. ^*^P < 0.05 and ^**^P < 0.01 compared with the control

Many studies have reported that the activation of NF-κB stimulated the expression of MMP-9 in various cancer cells [17]. Hence, we examined the effects of PLA on the activation of NF-κB signaling. We showed that treatment with 10 mM PLA for 45 min rapidly increased the phosphorylation levels of both IκBα and p65 in SiHa, HeLa, and C-33A cells (Fig. 4a–c). Because activation of NF-κB requires the translocation of the p65 subunit to the nucleus, the change in the levels of p65 in the cytoplasm and nucleus of PLA-treated HeLa and C-33A cells over time was determined. It is clear that PLA treatment substantially increased the levels of p65 in the nucleus of both HeLa and C-33A cells (Fig. 4d, e). Furthermore, the time-dependent increase in the luciferase activity of the NF-κB promoter verified the activation of NF-κB in HeLa and C-33A cells (Fig. 4f, g). These results implied that PLA increased the activation of NF-κB in cervical cancer cells.

### PLA increased MMP-9 expression through phosphorylation of IKK-β via PKC signals

**Fig. 5:**
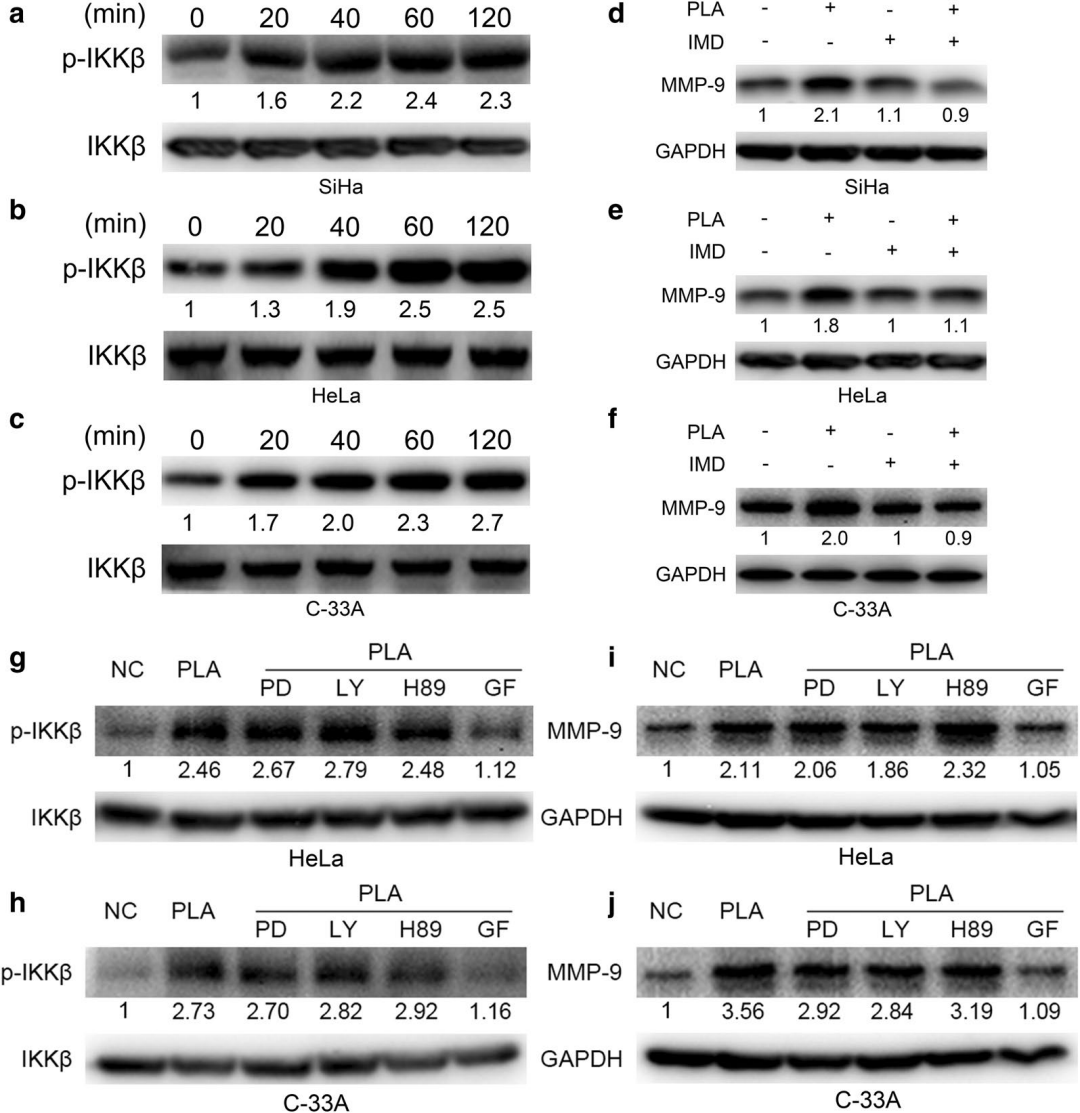
Effects of IKK-β on PLA-induced MMP-9 expression in cervical cancer cells. **a**‒**c** SiHa (**a**), HeLa (**b**), and C-33A (**c**) cells were treated with 10 mM PLA for the indicated times, and the activation of IKK-β was then examined by Western blot analysis. **d**‒**f** SiHa (**d**), HeLa (**e**), and C-33A (**f**) cells were pretreated with 10 μM IMD0354 (IMD, an inhibitor of IKK-β) for 90 min and then stimulated with 10 mM PLA for an additional 24 h. The expression of MMP-9 was measured by Western blot analysis. **g**‒**h** HeLa (**g**) and C-33A (**h**) cells were pretreated with 10 μM PD (ERK1/2 inhibitor), LY (PI3K/Akt inhibitor), H89 (PKA inhibitor), or GF (the inhibitor of PKC) for 90 min and then stimulated with 10 mM PLA for another 24 h. The activation of IKK-β were examined by Western blot analysis. **i**, **j** HeLa (**i**) and C-33A (**j**) cells were pretreated with 10 μM PD, LY, H89, or GF for 90 min, and then stimulated with 10 mM PLA for another 24 h. The expression of MMP-9 were examined by Western blot analysis. GAPDH expression was evaluated as a loading control. One representative of three different experiments, for each of the analyses performed, is shown

IκBα phosphorylation requires the constitutive activation of IKK-β protein [18]. We thus examined the effect of PLA on IKK activation. It was observed that PLA treatment significantly induced the activation of IKK-β in a time-dependent manner in SiHa, HeLa, and C-33A cells (Fig. 5a–c). To investigate whether activation of IKK-β is required for the PLA-stimulated upregulation of MMP-9, IKK-β was inactivated by treatment with its specific inhibitor IMD (hydrochloride). It was found that IMD significantly weakened the PLA-induced upregulation of MMP-9 in SiHa, HeLa, and C-33A cells (Fig. 5d–f). Therefore, the data suggested that IKK/NF-κB signaling mediates the PLA-induced increase in MMP-9 expression (Additional file 1: Fig. S3).

To find the direct target molecular responsible for the phosphorylation of IKK-β in PLA treated cervical cancer cells, we selected HeLa and C-33A cells and investigated several signals which were reported to modulate the biological behavior of cells, including PKA, ERK1/2, PI3K/Akt, and PKC, by using their specific inhibitors. The results shown that GF (the inhibitor of PKC) significantly reduced PLA induced phosphorylation of IKK-β (Fig. 5g, h) and up regulation of MMP-9 (Fig. 5i, j), while PD (ERK1/2 inhibitor), LY (PI3K/Akt inhibitor), or H89 (PKA inhibitor) had no such effect. Therefore, the results suggested that PLA increased the expression of MMP-9 by phosphorylation of IKK-β via the PKC signals.

### PLA mediated the activation of cervical cancer stemness

**Fig. 6:**
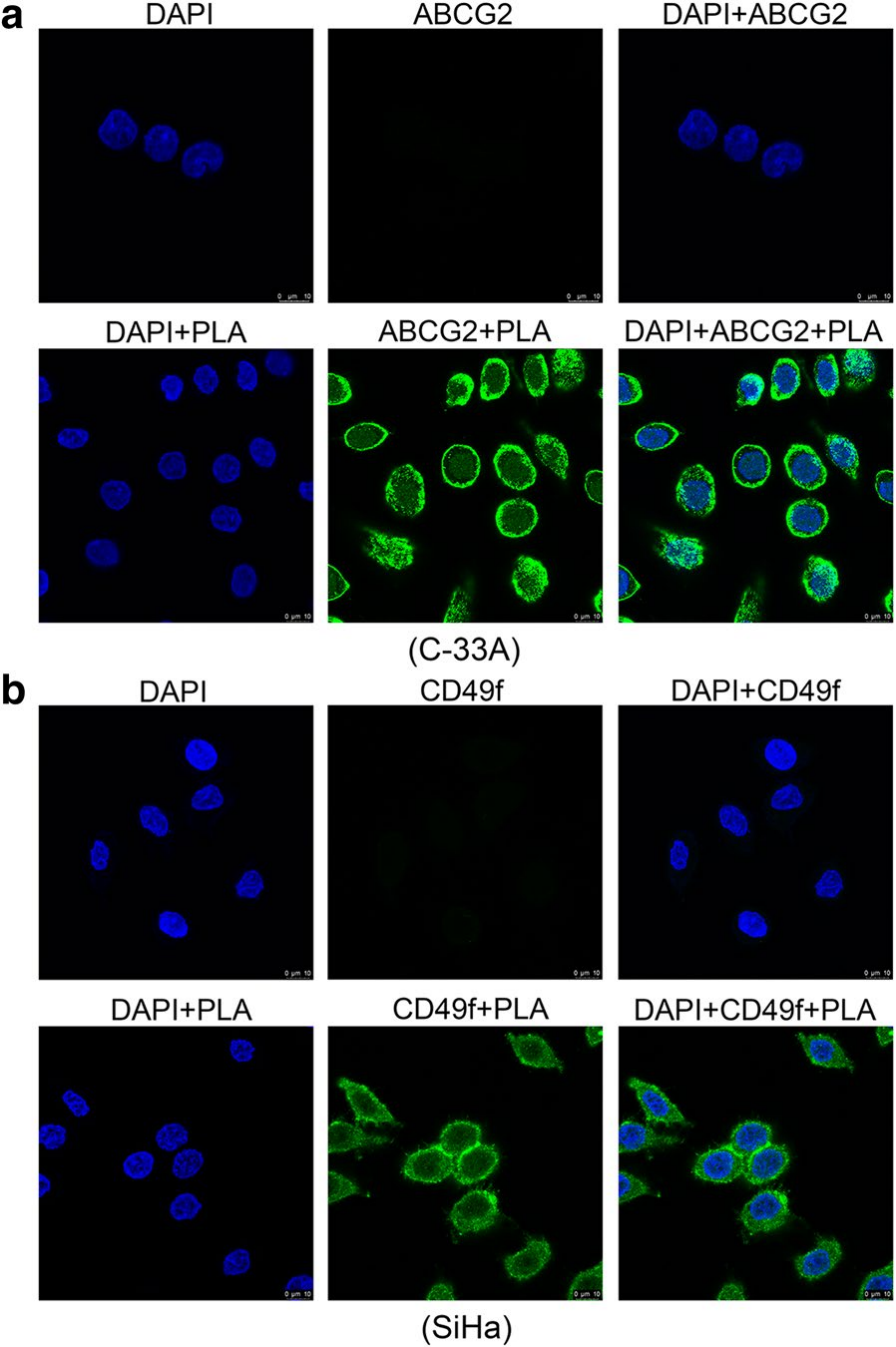
Fluorescence microscopy analysis of the labeled cervical cancer stemness markers. C-33A (**a**) and SiHa (**b**) cells were treated with or without PLA (10 mM) for 12 h, and then, ABCG2 and CD49f proteins were examined by immunostaining. DAPI (4′,6-diamidino-2-phenylindole) staining was applied as a nuclear marker. Scale bar, 10 μm

Cancer stem cells (CSCs) represent a small subpopulation of cells within a tumor that have properties of multilineage differentiation potential, self-renewal, slow cycling capacity, and tumorigenesis [19, 20]. Accumulating evidence implies that cervical CSCs (CCSCs) play crucial roles in the progression of cervical cancer, such as tumor metastasis and relapse [21]. Several markers, such as Oct4, ABCG2, ALDH1, SOX2 and CD49f, were identified as CCSC-specific markers in cervical cancer cell lines [22–24]. We therefore tested the expression of the five markers in PLA-treated or untreated SiHa, HeLa, and C-33A cells by using immunofluorescence staining. The results showed that PLA remarkably activated the expression of ABCG2 in C-33A cells and the expression of CD49f in SiHa cells (Fig. 6). The observed selective expression of CCSC-related markers in cervical cancer cells is also in accordance with that previously reported in multiple tumor types [23]. Therefore, the findings indicate that the PLA-induced activation of cervical cancer stemness populations might also be related to the elevated motility of cervical cancer cells in our study, suggesting that therapeutic treatment specifically targeting CCSCs may also be a useful tool for preventing tumor metastasis and recurrence in cervical cancer.

## Discussion

It is known that most deaths from cervical cancer occur as a consequence of metastasis rather than of the original tumor. Thus, it is crucial to uncover the mechanism underlying cancer cell metastasis. Prior research has suggested that elevated levels of lactic acid are related to tumor metastasis, recurrence and decreased patient survival [25]. Part of the mechanism of such pro-cancer behavior involves the promotion of cell migration and invasion through increasing the expression of microRNA-744 in SiHa cells [26]. In the current study, the capacity of PLA, another important organic acid produced by *Lactobacillus*, to affect the migration and invasion of cervical cancer cells was investigated. We found that PLA promoted the migration and invasion of SiHa, HeLa, and C-33A cells in vitro, which was accompanied by the upregulation of MMP-9 expression. Interestingly, PLA can also modulate the expression of HPV E6/E7 in SiHa and HeLa cells. Furthermore, we revealed for the first time that PLA significantly activated NF-κB signaling and triggered the nuclear translocation of NF-κB, which mediated PLA-induced cell migration and invasion as well as the regulation of E6/E7 and MMP-9 expression. Moreover, treatment with IMD, a specific inhibitor of IKK-β, abolished the PLA-induced upregulation of MMP-9. Therefore, our findings revealed a novel biological function of PLA as a tumor promoter in cervical cancer cells.

The NF-kB family is a family of important transcriptional factors known to regulate a wide range of biological effects. The activation of NF-κB is important for mediating cancer cell motility [27]. Constitutive NF-κB activation has been confirmed to be connected with tumor progression and aggressiveness, as well as poor prognoses, in breast, glioma, and cervical cancers [28]. Several studies have shown that inhibiting NF-κB activity can suppress cell migration, invasion, and metastasis by decreasing the expression of NF-κB downstream target genes, such as MMP-9, VEGF, uPA, and CXCR4 [29]. Conversely, the activation of NF-κB is significantly associated with progression to high-grade cervical squamous intraepithelial lesions and cervical cancer [30]. Several signaling pathways, such as the NF-κB, ERK1/2, PI3K/Akt, EGFR, and PKA pathways, have been indicated to function in cell migratory and invasive behavior [31]. However, our study demonstrated that only BAY 117082, an inhibitor of NF-κB, abolished PLA-induced cell migration and invasion via the downregulation of MMP-9 and the recovery of E6/E7 expression (Fig. 3). Furthermore, PLA treatment quickly increased the phosphorylation of both IκBα and p65 and induced the nuclear translocation and enhanced the promoter activity of NF-κB in the examined cervical cancer cells (Fig. 4). These results were in line with those of Spitkovsky et al. [32] whom showed that the E6 and E7 oncoproteins attenuated NF-κB activation by targeting the IκB kinases IKK-α and IKK-β.

An interesting observation is that the increases in cell migration and invasion were accompanied by the reduced expression of E6 and E7 in SiHa and HeLa cells. The phenomenon is similar to those performed in a recent study where lactic acid, the major organic acid secreted by lactic acid bacteria, down-regulated E6 and E7 protein levels and enhanced SiHa cell migration and invasion behavior [26]. Oncoproteins E6 and E7 are known to promote cancer cell motility through the modulation of different signaling pathways. For example, Zhu et al. demonstrated that E6/E7 upregulated MT1-MMP, MMP-2 and MMP-9 and promoted the migration of cervical cancer cells [33]. However, it is worth recalling that both E6 and E7 have been shown to inhibit the activation of critical antiviral transcription factors, such as NF-κB [34–36]. For instance, the E7 protein mediated the impaired nuclear translocation of cytoplasmic NF-κB by obviating IKK activation in the cytoplasm, while the E6 protein can conditionally inhibit NF-κB (p65)-dependent transcriptional activity within the nucleus; both of these effects result in attenuated NF-κB p65 activity [36, 37]. On the other hand, NF-κB, as a powerful repressor of HPV transcription, might in turn mediate a negative feedback loop to regulate hrHPV E6 and E7 expression. For example, NF-κB p65 was predominantly localized to the nucleus in siRNA (siE6 and/or siE7)-treated SiHa cells [38]. Herein, it was observed that PLA exerted a repressive effect on E6 and E7 expression (Fig. 1a), which might in turn have led to the activating effect on NF-κB (Fig. 4a). This result was similar to that of studies where E6 or E7 negatively interfered with NF-κB activity in human ovarian cancer, lung cancer, and osteosarcoma cells [32, 34, 39]. Therefore, considering the activation of NF-κB signaling and the concomitant appropriately acidic extracellular microenvironment induced by PLA (tumor acidity facilitates the invasive behavior of cancer cells), as well as our experimental results, it is conceivable that the inhibitory effect of decreased E6 and E7 expression on migration and invasion was negligible.

The invasion and metastasis of tumor cells is dependent on the degradation of the components of the extracellular matrix by MMPs, particularly MMP-2 and MMP-9 [15]. Earlier studies have indicated that NF-κB plays a significant role in activating MMP-2 and MMP-9. Overexpression of MMP-2 and MMP-9 has been frequently detected in solid tumors and has a direct effect on clinical outcome and prognosis [40]. Additionally, the secretion of MMP-2 and MMP-9 was demonstrated to be involved in the proteolytic events required for tumor migration and metastasis in human cervical cancer [41]. In agreement with previous work, we found that PLA-induced migration and invasion were accompanied by the upregulation of MMP-2 and MMP-9, MMP-1 and MMP-9, and MMP-2, MMP-9 and MMP-13 in SiHa, HeLa, and C-33A cells, while the expression of other MMPs was not significantly increased (Fig. 2). However, the different behaviors of MMP-1, MMP-2 and MMP-13 in the three cells could not be elucidated here because different culture media or state of HPV were used.

## Conclusion

In summary, this study has provided us with new information about PLA and its ability to function as a signaling molecule that modulates hrHPV E6/E7 expression and NF-κB signaling, thereby affecting both the migratory and invasive behavior of HPV16-positive SiHa, HPV18-positive HeLa, and HPV-negative C-33A cervical cells. It would be interesting to investigate in future studies whether PLA can inhibit the NF-κB signaling pathway in other cancers associated with HPV infection, such as anogenital, head and neck, and oropharyngeal cancers.

## Supplementary information

**Additional file 1: Table S1.** Primers used in this study. **Fig. S1.** Effects of PLA on the proliferation of cervical cancer cells. (a) SiHa. (b) HeLa. (c) C-33A. Cells were treated with 2.5 to 20 mM PLA. Cell proliferation was documented every 24 h for 3 days using the colorimetric MTT assay (Sigma, St. Louis, MO), and absorbance at 490 nm was evaluated by a Spectra Max 190 microplate reader (Molecular Devices, Sunnyvale, CA). Data are presented as the means ± SDs of three independent experiments. ^*^P < 0.05 and ^**^P < 0.01 compared with the control. **Fig. S2.** Effects of NF-κB on the PLA-induced migration and invasion of cervical cancer cells. SiHa, HeLa, and C-33A cells were pretreated with 10 μM PD98059 (PD, a specific antagonist of ERK1/2 kinase), LY294002 (LY, a selective antagonist of PI3 K/Akt), AG1478 (AG, a potent antagonist of EGFR), H89 (H89, a selective antagonist of PKA), and BAY11-7082 (BAY, an inhibitor of NF-κB) for 90 min without PLA treatment. Cell migration (a, c, e) and invasion (b, d, f) were monitored by Transwell assays. The number of migrated and invaded cells was obtained by comparison with the control cells. Data are presented as the means ± SDs of three independent experiments. ^*^P < 0.05 and ^**^P < 0.01 compared with the control. **Fig. S3.** Construction of stable IKKβ knockdown HeLa and C-33A cell lines to validate the pathway PLA undergoes. (a, d) Stable HeLa (a) and C-33A (d) cells were established by lentivirus infection with scrambled shRNA (shCtrl) and 2 specific shRNAs against IKKβ (shIKKβ#1 and shIKKβ#2). Cells lysates were subjected to Western blot analysis with the indicated antibodies. (b, e) Proliferation of the HeLa (b) and C-33A (e) stable cell lines was detected by MTS assay. The cell proliferation index of the shCtrl group at 24 h was defined as 100%. (c, f) Western blot analysis of MMP-9 protein levels in the stable HeLa (c) and C-33A (f) cell lines treated with or without PLA. GAPDH expression was evaluated as a loading control. One representative of three different experiments, for each of the analyses performed, is shown. Data are presented as the means ± SDs of three independent experiments. ^*^P < 0.05, ^**^P < 0.01, and ^***^P < 0.001 compared with the control. **Methods S1.** Lentiviral construction and cell infection. shRNAs (shIKKβ#1 and shIKKβ#2) against human IKKβ and a negative control (shCtrl) were synthesized by Shanghai GeneChem Co., Ltd. (Shanghai, China) and then annealed and ligated into the pGCSIL-GFP vector.

## Data Availability

All data generated or analyzed during the present study are included in this published article.

## Abbreviations

hrHPV: high-risk human papillomavirus
PLA: phenyllactic acid
MMP: matrix metalloproteinase
NF-κB: nuclear factor-kappa B
IKK-β: IκB kinase-β
CSCs: cancer stem cells
CCSCs: cervical cancer stem cells
