# Supplementary material for: Phenyllactic acid promotes cell migration and invasion in cervical cancer via IKK/NF-κB-mediated MMP-9 activation
Source: Cancer Cell Int. 2019 Sep 23;19:241. doi: 10.1186/s12935-019-0965-0 (PMC6757389; doi:10.1186/s12935-019-0965-0)
Supplement: Supplementary file 1 — Additional file 1: Table S1. Primers used in this study. Fig. S1. Effects of PLA on the proliferation of cervical cancer cells. (a) SiHa. (b) HeLa. (c) C-33A. Cells were treated with 2.5 to 20 mM PLA. Cell proliferation was documented every 24 h for 3 days using the colorimetric MTT assay (Sigma, St. Louis, MO), and absorbance at 490 nm was evaluated by a Spectra Max 190 microplate reader (Molecular Devices, Sunnyvale, CA). Data are presented as the means ± SDs of three independent experiments. *P < 0.05 and **P < 0.01 compared with the control. Fig. S2. Effects of NF-κB on the PLA-induced migration and invasion of cervical cancer cells. SiHa, HeLa, and C-33A cells were pretreated with 10 μM PD98059 (PD, a specific antagonist of ERK1/2 kinase), LY294002 (LY, a selective antagonist of PI3 K/Akt), AG1478 (AG, a potent antagonist of EGFR), H89 (H89, a selective antagonist of PKA), and BAY11-7082 (BAY, an inhibitor of NF-κB) for 90 min without PLA treatment. Cell migration (a, c, e) and invasion (b, d, f) were monitored by Transwell assays. The number of migrated and invaded cells was obtained by comparison with the control cells. Data are presented as the means ± SDs of three independent experiments. *P < 0.05 and **P < 0.01 compared with the control. Fig. S3. Construction of stable IKKβ knockdown HeLa and C-33A cell lines to validate the pathway PLA undergoes. (a, d) Stable HeLa (a) and C-33A (d) cells were established by lentivirus infection with scrambled shRNA (shCtrl) and 2 specific shRNAs against IKKβ (shIKKβ#1 and shIKKβ#2). Cells lysates were subjected to Western blot analysis with the indicated antibodies. (b, e) Proliferation of the HeLa (b) and C-33A (e) stable cell lines was detected by MTS assay. The cell proliferation index of the shCtrl group at 24 h was defined as 100%. (c, f) Western blot analysis of MMP-9 protein levels in the stable HeLa (c) and C-33A (f) cell lines treated with or without PLA. GAPDH expression was evaluated as a loading control. One [file 12935_2019_965_MOESM1_ESM.docx]

**Table S1** Primers used in this study.

| Gene | Primer name | Sequence (5' to 3') |  |
| --- | --- | --- | --- |
| HPV16 E6 | 16E6-F | GACCCAGAAAGTTACCACAG | |
|  | 16E6-R | CATAAATCCCGAAAAGCAAAG | |
| HPV16 E7 | 16E7-F | GGAGGAGGATGAAATAGATGG | |
|  | 16E7-R | TGAGAACAGATGGGGCACAC | |
| HPV18 E6 | 18E6-F | GTATGGAGACACATTGGAA | |
|  | 18E6-R | CTGGATTCAACGGTTTCT | |
| HPV18 E7 | 18E7-F | CAATTAAGCGACTCAGAG | |
|  | 18E7-R | TTACAACACATACACAACAT | |
| GAPDH | GAPDH-F | CTGCACCACCAACTGCTTAG | |
|  | GAPDH-R | TTCTGGGTGGCAGTGATG | |
| MMP-1 | MMP1-F | GGGGCTTTGATGTACCCTAGC | |
|  | MMP1-R | TGTCACACGCTTTTGGGGTTT | |
| MMP-2 | MMP2-F | TACAGGATCATTGGCTACACACC | |
|  | MMP2-R | GGTCACATCGCTCCAGACT | |
| MMP-3 | MMP3-F | AGTCTTCCAATCCTACTGTTGCT | |
|  | MMP3-R | TCCCCGTCACCTCCAATCC | |
| MMP-9 | MMP9-F | TGTACCGCTATGGTTACACTCG | |
|  | MMP9-R | GGCAGGGACAGTTGCTTCT | |
| MMP-10 | MMP10-F | TGCTCTGCCTATCCTCTGAGT | |
|  | MMP10-R | TCACATCCTTTTCGAGGTTGTAG | |
| MMP-13 | MMP13-F | ACTGAGAGGCTCCGAGAAATG | |
|  | MMP13-R | GAACCCCGCATCTTGGCTT | |


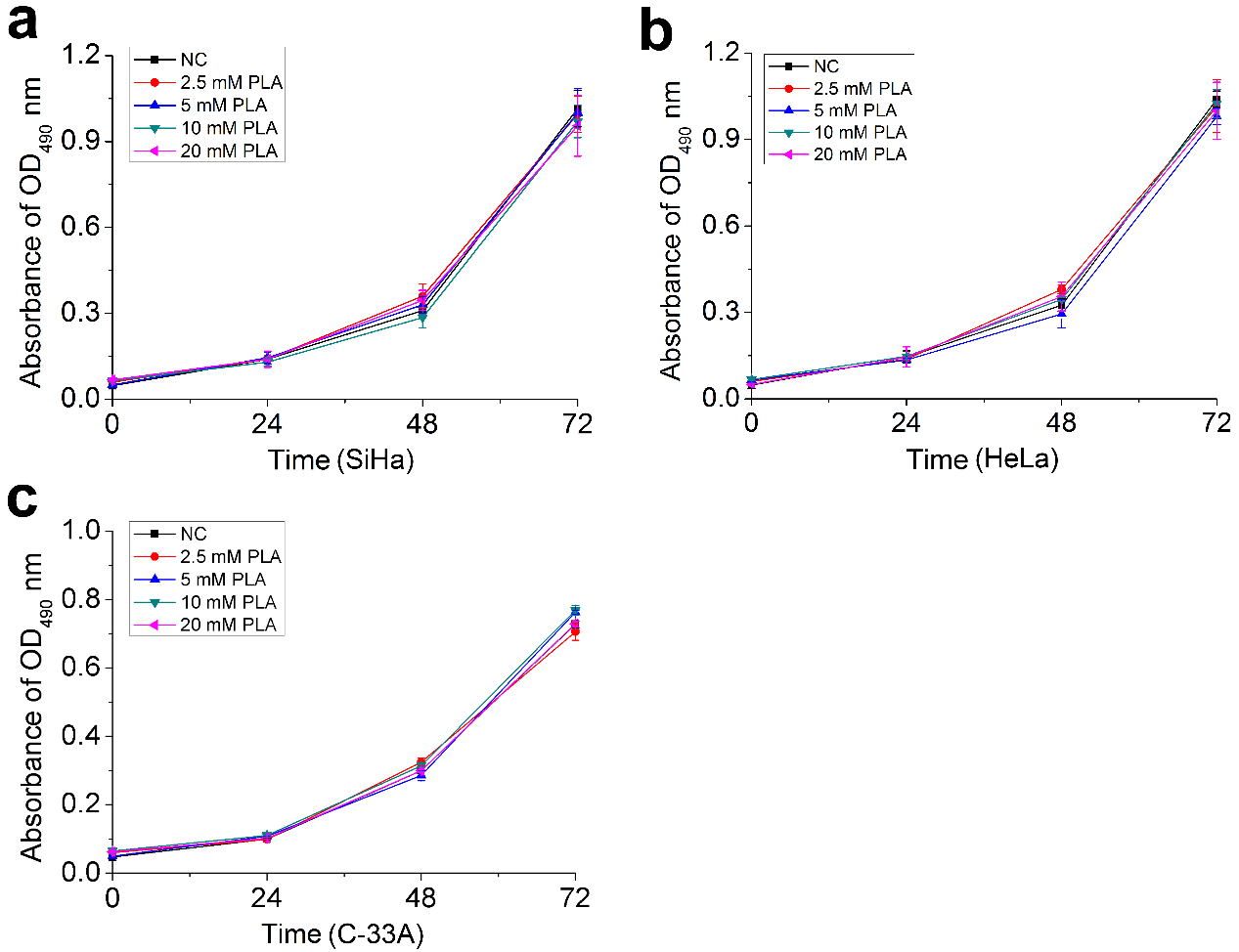


**Fig. S1.** Effects of PLA on the proliferation of cervical cancer cells. (a) SiHa. (b) HeLa. (c) C-33A. Cells were treated with 2.5 to 20 mM PLA. Cell proliferation was documented every 24 h for 3 days using the colorimetric MTT assay (Sigma, St. Louis, MO), and absorbance at 490 nm was evaluated by a Spectra Max 190 microplate reader (Molecular Devices, Sunnyvale, CA). Data are presented as the means ± SDs of three independent experiments. ^*^*P* < 0.05 and ^**^*P* < 0.01 compared with the control.


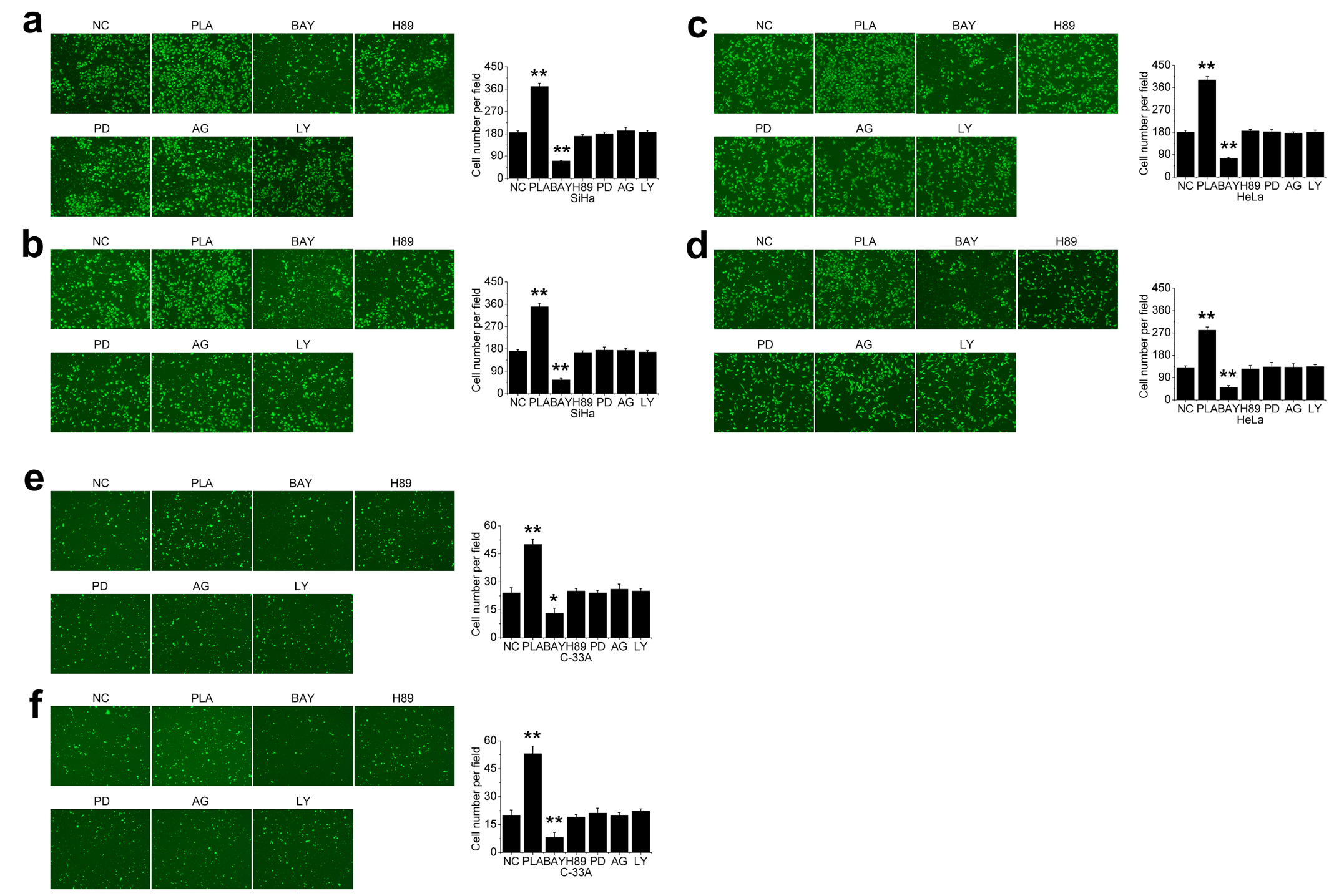


**Fig. S2.** Effects of NF-κB on the PLA-induced migration and invasion of cervical cancer cells. SiHa, HeLa, and C-33A cells were pretreated with 10 μM PD98059 (PD, a specific antagonist of ERK1/2 kinase), LY294002 (LY, a selective antagonist of PI3K/Akt), AG1478 (AG, a potent antagonist of EGFR), H89 (H89, a selective antagonist of PKA), and BAY11-7082 (BAY, an inhibitor of NF-κB) for 90 min without PLA treatment. Cell migration (a, c, e) and invasion (b, d, f) were monitored by Transwell assays. The number of migrated and invaded cells was obtained by comparison with the control cells. Data are presented as the means ± SDs of three independent experiments. ^*^*P* < 0.05 and ^**^*P* < 0.01 compared with the control.


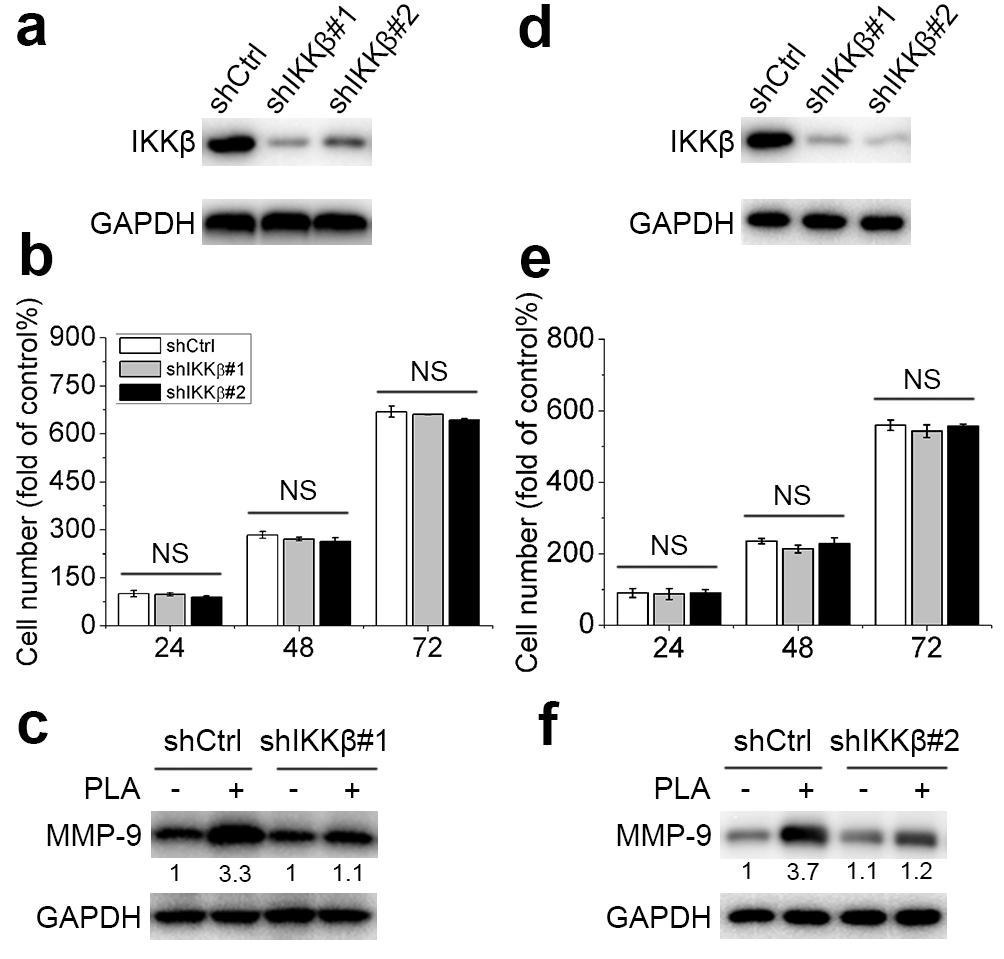


**Fig. S3.** Construction of stable IKKβ knockdown HeLa and C-33A cell lines to validate the pathway PLA undergoes. (a, d) Stable HeLa (a) and C-33A (d) cells were established by lentivirus infection with scrambled shRNA (shCtrl) and 2 specific shRNAs against IKKβ (shIKKβ#1 and shIKKβ#2). Cells lysates were subjected to Western blot analysis with the indicated antibodies. (b, e) Proliferation of the HeLa (b) and C-33A (e) stable cell lines was detected by MTS assay. The cell proliferation index of the shCtrl group at 24 h was defined as 100%. (c, f) Western blot analysis of MMP-9 protein levels in the stable HeLa (c) and C-33A (f) cell lines treated with or without PLA. GAPDH expression was evaluated as a loading control. One representative of three different experiments, for each of the analyses performed, is shown. Data are presented as the means ± SDs of three independent experiments. ^*^*P* < 0.05, ^**^*P* < 0.01, and ^***^*P* < 0.001 compared with the control.

[**Methods S1:** Lentiviral construction and cell infection. shRNAs (shIKKβ#1 and shIKKβ#2) against human IKKβ and a negative control (shCtrl) were synthesized by Shanghai GeneChem Co., Ltd. (Shanghai, China) and then annealed and ligated into the pGCSIL-GFP vector. A human IKKβ knockdown stable cell line was constructed according to a previous study[Li, 2011 #2539] (Boko Haram. *Encyclopaedia Britannica*. Encyclopædia Britannica, Inc;2014.).]
